# Role of BMI in the Relationship Between Dietary Inflammatory Index and Depression: An Intermediary Analysis

**DOI:** 10.3389/fmed.2021.748788

**Published:** 2021-11-15

**Authors:** Yuxia Ma, Ruiqiang Li, Wenqiang Zhan, Xin Huang, Zechen Zhang, Shuaishuai Lv, Jiaqi Wang, Luyao Liang, Xiaofang Jia

**Affiliations:** ^1^Department of Nutrition and Food Hygiene, School of Public Health, Hebei Medical University, Hebei Province Key Laboratory of Environment and Human Health, Shijiazhuang, China; ^2^School of Public Health, Shanghai Jiao Tong University School of Medicine, Shanghai, China; ^3^National Institute for Nutrition and Health, Chinese Center for Disease Control and Prevention, Beijing, China

**Keywords:** dietary inflammatory index, depression, body mass index, mediation analysis, diet

## Abstract

**Introduction:** This study investigated this association and the role of BMI in the inflammatory process in a large population-based observational study.

**Methods:** A total of 1,865 elderly people (≥55 years) were followed from the Community Cohort Study of Nervous System Diseases (CCSNSD) cohort study from 2018 to 2019 (Mean [SD] age, 66.31 [0.32] years; 716 [38.4%] males). The semi-quantitative FFQ and geriatric depression scale (GDS) were used to evaluate the diet and depressive symptoms of the elderly, respectively. The multivariable logistic regression model estimated the OR and 95% CI between Empirical Dietary Inflammatory Index (E-DII) and depression. The interaction of E-DII and BMI on depressive events was tested, and the mediation analysis of BMI was performed.

**Results:** As measured by E-DII, the mean (SE) value of the inflammatory potential of the diet in our study was 1.56 (0.12). E-DII ranged from 5.23 to 5.58. In comparison with the first quartile, the elderly from the second quartile (OR: 1.15 [95% CI: 1.09, 1.28]) to the fourth quartile (OR: 1.31 [95% CI: 1.16, 1.42]) have a higher risk of depression before adjustment for BMI. An interaction was observed between E-DII and BMI in terms of the risk of depression (P_Interaction_ < 0.001). The whole related part is mediated by BMI (31.06%).

**Conclusion:** Our findings indicate that the higher pro-inflammatory potential of diet is associated with a higher risk of depression, and this association may be mediated by BMI. Further research is needed to verify our findings and clarify the latent mechanism.

## Introduction

Depression is an increasingly serious public health problem and the main cause of disability worldwide, especially among the elderly (1). Approximately 18% of adults aged 65 and over suffer from depression in England (2). Depression in later life is associated with a wide range of adverse health outcomes, including the risk of morbidity, cognitive decline, and increased mortality (3–5). However, the exact biological mechanism of the pathogenesis of depression remains unknown (6).

Inflammation plays a key role in the pathogenesis of depression. Obesity, hyperglycemia, insulin resistance, and overexpression of pro-inflammatory proteins, such as C-reactive protein and cytokines (IL-1β, IL-6, and TNF-α), can induce chronic inflammation of depression (7). The association between inflammation and mental illness is described as a two-way comorbidity, which means that increased inflammation of the body is associated with an increased risk of mental problems (such as depression), and depression itself is associated with increased behaviors that trigger inflammation, such as Unhealthy eating habits (8). In addition, environmental, behavioral, and psychosocial factors can stimulate inflammation during stress. Among the modifiable factors, diet is a key lifestyle-related factors that can regulate the inflammatory process (9, 10). Several foods and food components affect the blood concentration of inflammatory markers, including cytokines, chemokines, acute phase proteins, soluble adhesion molecules, and cytokine receptors (11).

Recently, the dietary inflammatory index (DII) was developed based on evidence linking diet to inflammation. Limited studies have evaluated the link between these indicators and depression, and the results are conflicting (12, 13). In addition, the association between DII and depression may be partly mediated by BMI (12). Thus, BMI has an intermediate role in the association between DII and depression, but the current role of BMI between DII and depression is still ambiguous. Empirical Dietary Inflammatory Index (E-DII) is an updated development of DII, representing a novel, hypothesis-driven, and empirically derived diet pattern that can be used to evaluate diet quality based on its inflammatory potential. It has strong construct validity in independent samples of women and men, indicating that it is useful in assessing the inflammatory potential of the entire diet. In addition, EDII can be calculated in a standardized and reproducible manner among different populations, thereby circumventing the main limitations of dietary patterns derived from the same study that applied them (14). Therefore, based on data from the Community Cohort Study of Nervous System Diseases CCSNSD) cohort study, the present study aimed to prospectively assess the association between E-DII and depression and to evaluate potential interactions and the mediating role of BMI in this relationship.

## Materials and Methods

### Study Population

Participants in the current study were derived from the baseline of the Community Cohort Study of Nervous System Diseases (CCSNSD), an ongoing longitudinal study established by the project in 2018-2019, focusing on potential factors related to the risk of three neurological diseases, including epilepsy in patients >1-year-old and Alzheimer's disease (AD), Parkinson's disease (PD) in people ≥55 years old. The project is undertaken by the Institute of Nutrition and Health of the Chinese Center for Disease Control and Prevention, in cooperation with the Center for Disease Control and Prevention. The project applies a multistage random cluster sampling method to extract samples. The protocol of this study was reviewed and approved by the Institutional Review Board of the National Institute for Nutrition and Health (No. 2017020, November 6, 2017). In addition, written informed consent was obtained for each participant before the survey (10).

**Figure 1:**
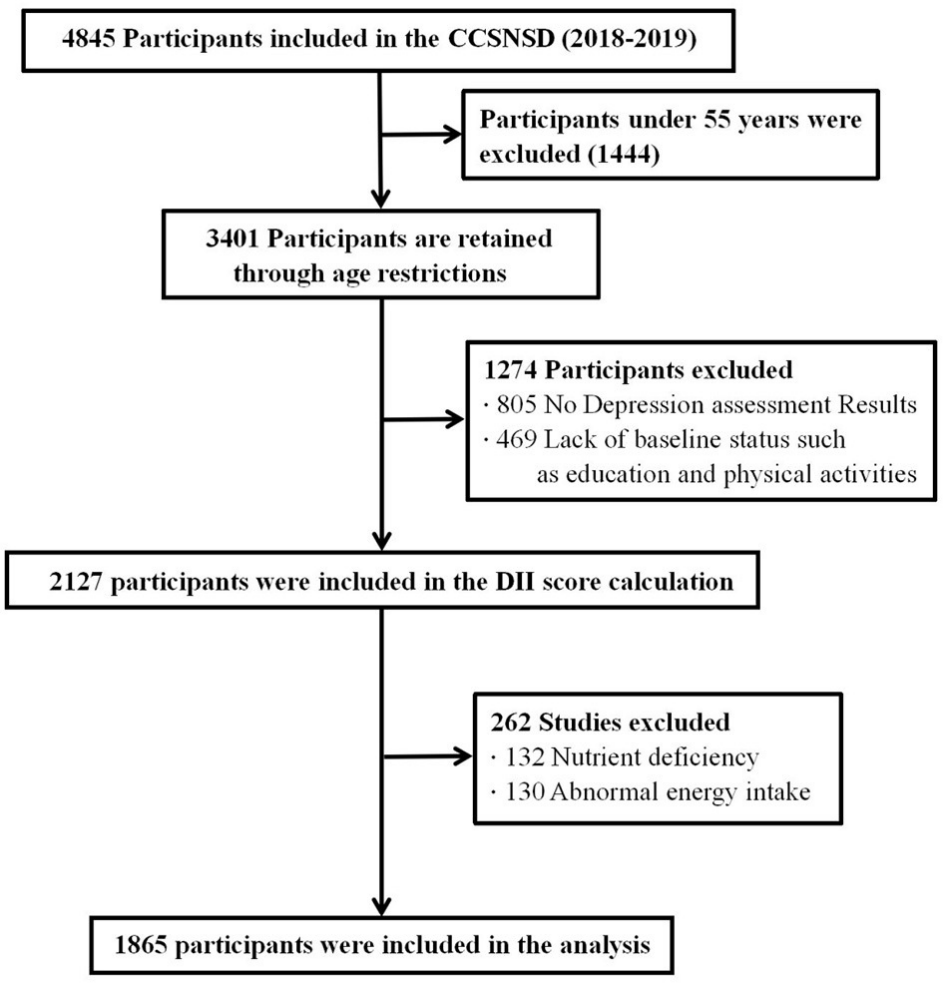
Selection process of subjects.

The samples eligible for inclusion were (1) 55 years old and older, (2) resident population living in the sampled community, (3) patients not clinically diagnosed with depression, (4) ability to perform a normal depression assessment, and (5) completed data of sociodemographic characteristics, disease history, and FFQ. We excluded subjects because of (1) absence of depression assessment results, (2) lack of baseline status such as education and physical activities, (3) nutrient deficiency, and (4) abnormal energy intake. Finally, a total of 1,865 participants were involved in the analysis (shown in Figure 1).

### Depression

We defined depression according to the geriatric depression scale (GDS), which is widely used scale to assess depression symptoms among the elderly (15). It consists of 30 self-assessment items with yes/no response options. A score of 0–10 indicates no depression symptoms, 11–20 indicates mild depression, and 21–30 indicates severe depression symptoms (16).

### Dietary Measurements

A previously validated 116 semi-quantitative food frequency questionnaire (FFQ) (24) was used to assess diet. For each item, participants were asked to specify how often they consumed food or beverages on average in the previous year. The frequency of consumption response is divided into never, < once/month, 1–3 times/month, once/week, 2–4 times/week, 5–6 times/week, once/day, 2–3 times /day, 4–5 times/day, and ≥6 times/day. Energy and nutrient intake was calculated by multiplying the frequency of consumption per unit of food by the energy and nutrient content of a specified serving. The composition value of energy and nutrients was calculated using the Chinese food content database.

### Dietary Inflammatory Index

E-DII was developed by Shivappa et al. In short, a systematic review of all publications that assess the relationship between inflammatory markers and dietary factors was used as the basis for formulating E-DII. Based on literature, 45 food parameters are always associated with inflammation markers (positive or negative). Finally, for each food parameter, a specific inflammatory effect score was assigned according to the number of studies reporting anti-inflammatory, pro-inflammatory, or non-inflammatory effects; then, the scores were weighted by the study design and the number of articles related to each diet parameter/inflammatory marker. More information about E-DII is available elsewhere (17).

In our study, E-DII was calculated using baseline FFQ data, which captured 22 of the 45 possible foods and nutrients in the original E-DII. The 22 parameters include carbohydrates, protein, fat, β-carotene, fiber, cholesterol, saturated fat, monounsaturated fat, polyunsaturated fats, niacin, thiamine, riboflavin, vitamin B12, vitamin B6, iron, magnesium, zinc, selenium, vitamin A, vitamin C, vitamin E, and folic acid. To obtain E-DII, we first estimated the intake of each food through FFQ. Then, we used a representative global database to obtain the standardized intake, which was then converted into percentile score. To obtain a symmetrical distribution centered on 0, we multiplied the percentile score by 2 and subtracted 1. Then, we multiplied the percentile score by the inflammatory effect score, as defined by “Food parameter specific E-DII score”. Finally, through the energy density correction, all food parameters were added together to obtain E-E-DII. A lower E-E-DII score indicates an anti-inflammatory diet, while a higher E-E-DII score indicates a pro-inflammatory diet. For our analysis, 22 food parameters were used, and the score ranged from −5.23 and 5.28. We converted continuous variables into quartiles (Q), including highly anti-inflammatory (Q1: −5.23, 0.79), anti-inflammatory (Q2: 0.80, 1.80), inflammation (Q3: 1.81, 2.54), and high inflammation (Q4: 2.55, 5.28).

### Covariates

We adjusted for variables previously identified as potential confounders in the literature. The included baseline characteristics include self-reported age (years), BMI (<18.5 kg/m^2^, 18.50–23.9 kg/m^2^, 24–28 kg/m^2^, and ≥28 kg/m^2^), gender (female or male), education level (illiterate, elementary school, junior high school, and above), employment status (yes or no), lifestyle and health-related variables including smoking (yes or no), drinking (yes or no), physical activity (yes or no), daily energy intake (kcal), diabetes (yes or no), and high blood pressure (yes or no). The missing values of all variables are less than 5%. Hence, the median and mode of quantitative and qualitative variables were used for inference.

### Statistical Analyses

The baseline characteristics of the study population were reported as the mean (Standard error, SEM) of continuous variables and the number (percentage) of categorical variables. We used the logistic proportional hazards model to estimate OR and 95% CI.

E-E-DII was modeled in three different ways. For our main analysis, E-E-DII was included as a continuous variable, and we reported an estimate of the effect of 1-SD increase (z-score) in E-DII. This method assumes a linear relationship between E-DII and depression. To check the hypothesis, we then used a multivariate restriction cubic spline and placed three nodes at the 25th, 50th, and 75th percentile of the E-DII distribution nodes to provide a graphical representation. The spline curve allowed us to test whether for significant difference with the linear correlation. Finally, we divided the E-DII into quarters and considered the first quarter group as the reference category in the logistic model.

Three models were proposed: the first model did not adjust for confounding factors; the second model was further adjusted for known risk factors for depression and potential confounding factors, such as age, sex, physical activity, education level, smoking status, drinking status, daily energy intake, and employment status; and The third model further adjusted BMI. The confounding factors were selected *a priori* based on known depression risk factors available in our data set and related to E-DII.

The interaction of E-DII and BMI on the risk of depression was estimated by including a multiplication term between the two variables in the logistic model. Considering the statistical significance, we examined the association between E-DII and depression stratified by BMI category.

Regression-based mediation analysis was used to distinguish the direct effect of adherence to E-DII on the risk of depression and the indirect effect mediated by BMI. Three estimates were obtained as follows:

- Total effect, that is, the overall association between E-DII and the risk of depression, including the association mediated by BMI;
- Direct effect, that is, the association between E-DII and depression risk, adjusted according to BMI; and
- Indirect effect, that is, the association between E-DII and depression risk, are mediated by BMI.

In addition, to effectively understand the complex relationship between E-DII, BMI, and depression, we used a counterfactual mediation model that allows exposure-medium interaction. According to this model, BMI represents the relationship between E-DII and depression (18–20).

All statistical analyses were performed using the software package R (http://www.R-project.org, The R Foundation). A two-tailed *p*-value of <0.05 was considered statistically significant.

## Results

### Baseline Characteristics

As measured by E-DII, the mean (SEM) value of the inflammatory potential of the diet in our cohort was 1.56 (0.12). E-DII ranged from 5.23 to 5.58. The median and 25th and 75th percentiles are 0.79, 1.80, and 2.54, respectively. A total of 447 (24.0%) verified cases of depression symptoms were identified. The population mean (SE) age at baseline was 66.31 years (0.32). In general, as the E-DII quartile increases, the average age, BMI, and frequency of depression cases increase (Table 1).

**Table 1:** Baseline characteristics of the study population(CCSNSD cohort data, *N* = 1865).

**Characteristic**	**Frequency (%) or Mean (SEM)**				***P*–value**
	**Quartile 1 (*n =* 467)**	**Quartile 2 (*n =* 468)**	**Quartile 3 (*n =* 464)**	**Quartile 4 (*n =* 466)**	
Age (years)					<0.001
	68.62 ± 0.33	66.70 ± 0.34	64.50 ± 0.32	65.39 ± 0.33	
Sex					0.912
Male	184 (39.4%)	174 (37.2%)	180 (38.8%)	178 (38.2%)	
Female	283 (60.6%)	294 (62.8%)	284 (61.2%)	288 (61.8%)	
BMI (kg/m2)					0.018
BMI <18.50 (underweight)	14 (3.0%)	15 (3.2%)	13 (2.8%)	13 (2.8%)	
18.5 ≤ BMI <24.00 (normal weight)	190 (40.7%)	181 (38.7%)	168 (36.2%)	161 (34.5%)	
24.00 ≤ BMI <28.00 (overweight)	181 (38.8%)	185 (39,5%)	193 (41.6%)	195 (41.8%)	
BMI≥28.00 (obese)	82 (17.6%)	87 (18.6%)	90 (19.4%)	97 (20.8%)	
Employment					0.475
No	412 (88.2%)	403 (86.1%)	393 (84.7%)	401 (86.1%)	
Yes	55 (11.8%)	65 (13.9%)	71 (15.3%)	65 (13.9%)	
Education					0.038
Illiteracy	119 (25.5%)	136 (29.1%)	104 (20.4%)	112 (24.0%)	
Primary school	164 (35.1%)	156 (32.3%)	140 (30.2%)	146 (31.3%)	
Junior high school/above	184 (39.4%)	176 (37.6%)	220 (47.4%)	208 (44.6%)	
Daily energy intake (kcal)					<0.001
	1,534.67 ± 16.21	1,361.06 ± 15.55	1,252.09 ± 14.66	1,329.95 ± 15.52	
Tobacco Smoking					0.594
No	386 (82.7%)	398 (85.0%)	398 (85.8%)	393 (84.3%)	
Yes	81 (17.3%)	70 (15.0%)	66 (14.2%)	73 (15.7%)	
Alcohol Drinking					0.671
No	427 (91.4%)	420 (89.7%)	424 (91.4%)	418 (89.7%)	
Yes	40 (8.6%)	48 (10.3%)	40 (8.6%)	48 (10.3%)	
Physical activities					0.872
No	279 (59.7%)	289 (61.8%)	280 (60.3%)	289 (62.0%)	
Yes	188 (40.3%)	179 (38.2%)	184 (39.7)	177 (38.0%)	
Hypertension					0.821
No	188 (40.3%)	197 (42.1%)	189 (40.7%)	182 (39.1%)	
Yes	279 (59.7%)	271 (57.9%)	275 (59.3%)	284 (60.9%)	
Diabetes					0.886
No	393 (84.2%)	394 (84.2%)	384 (82.8%)	394 (84.5%)	
Yes	74 (15.8%)	74 (15.8%)	80 (17.2%)	72 (15.5%)	
Depression					0.002
No	369 (79.0%)	358 (76.5%)	348 (75.0%)	343 (73.6%)	
Yes	98 (21.0%)	110 (23.5%)	116 (25.0%)	123 (26.4%)	

*Data are N (%) for categorical variables. Data are mean (SEM) for continuous variables*.

### E-DII and Depression

**Figure 2:**
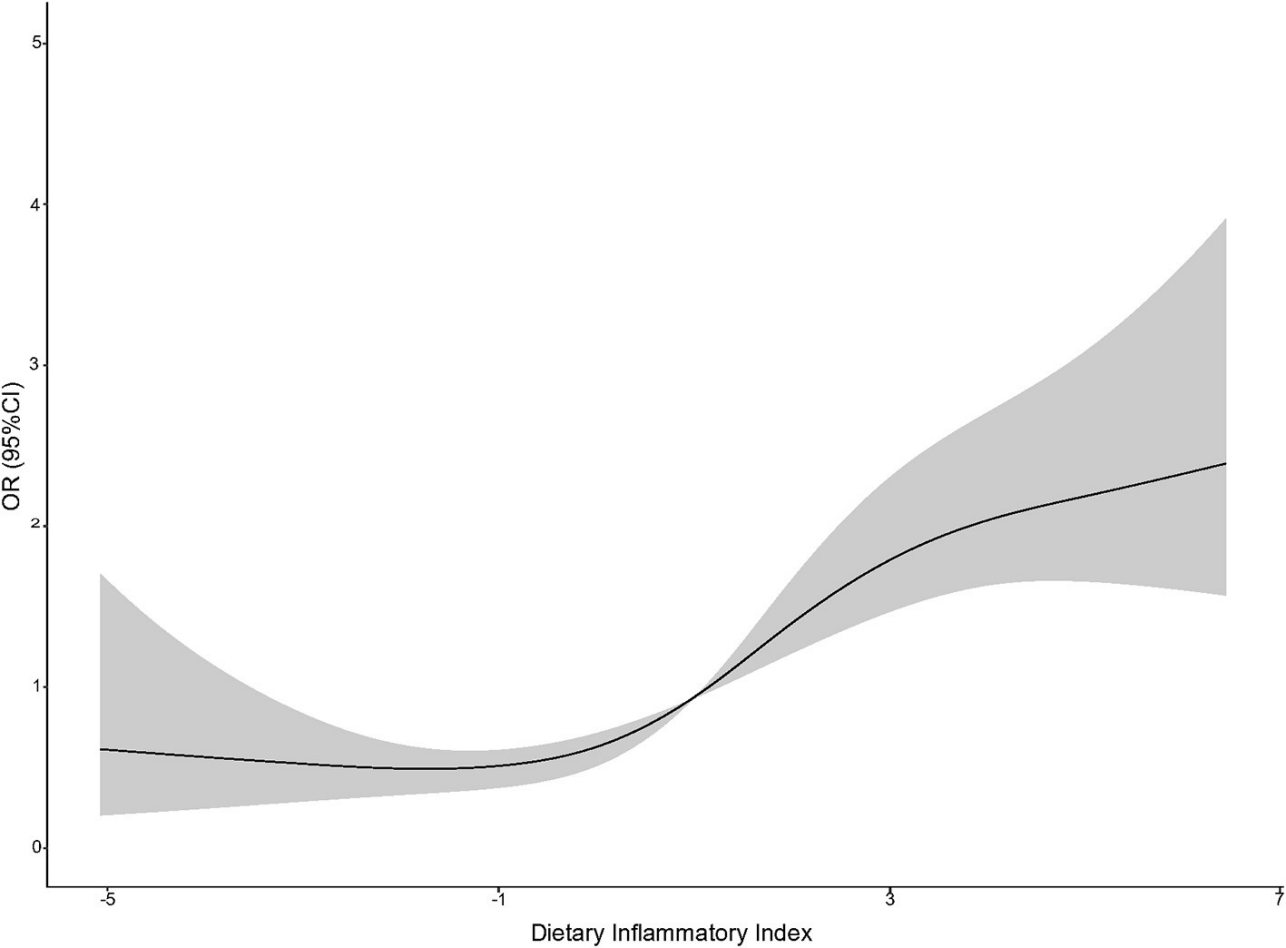
A restricted cubic spline curve was used to fit the relationship between DII and the risk of depression (the three nodes are located at the 25th, 50th, and 75th percentiles). Risk estimates were adjusted for age, sex, physical activity, education level, smoking status, drinking status, daily energy intake, and employment status. Reference value for ORs: median score (1.80); the solid black line represents the OR and the shaded part the lower and upper 95% CI; *P*_Overall_ <0.001; *P*_Nonlinear_ = 0.009.

The association between E-DII and depression is shown in Table 2. In the first two models, E-DII was positively correlated with the risk of depression in the second quartile (OR: 1.15 [95% CI: 1.09, 1.28]) to the fourth quartile (OR: 1.31 [95% CI: 1.16, 1.42]) compared with the first quartile. An analysis with E-DII to increase 1-SD yielded similar results (Model 2: OR: 1.13 [95% CI: 1.06, 1.22]). The spline variable confirmed that E-DII was non-linearly associated with the risk of depression (*P* = 0.009), and the graph of the relationship showed that the increase in E-DII was accompanied by increased risk of depression (shown in Figure 2). After being included in BMI (Model 3), all associations weakened and became invalid (*P* > 0.05) (Table 2).

**Table 2:** Risk of depression according to quartile groups of E–DII (*N* = 1865).

	**Participants without depression N (%)**	**Participants with depression N (%)**	**Model 1**	**Model 2**	**Model 3**
			**OR (95% CI)**	**OR (95% CI)**	**OR (95% CI)**
For 1–SD increase	*N =* 1418	*N =* 447	1.12 (1.08–1.25)	1.13 (1.06–1.22)	1.08 (0.98–1.22)
**Quartile groups of E–DII**					
Q1	369 (79.0%)	98 (21.0%)	Reference		
Q2	358 (76.5%)	110 (23.5%)	1.15 (1.09–1.29)	1.15 (1.09–1.28)	1.12 (0.82–1.21)
Q3	348 (75.0%)	116 (25.0%)	1.20 (1.12–1.31)	1.23 (1.12–1.35)	1.18 (0.96–1.28)
Q4	343 (73.6%)	123 (26.4%)	1.26 (1.15–1.38)	1.31 (1.16–1.42)	1.25 (0.98–1.36)
P–trend			0.001	0.006	0.286

*Model 1: unadjusted. Model 2: age, sex, physical activity, education level, smoking status, drinking status, hypertension, diabetes, daily energy intake, employment status. Model 3: further adjusted for BMI*.

### E-DII and Depression Risk Stratified by BMI Category

Table 3 shows the association between E-DII and depression stratified by BMI category. An interaction was observed between E-DII and BMI on the risk of depression (*P*_Interaction_ < 0.001). When stratified by BMI category, the risk of E-DII and depression was mainly reflected in obese people (model 2: OR: 1.27 [95% CI: 1.12, 1.42], fourth quartile of E-DII vs. first quartile), No significant association was observed in other populations.

**Table 3:** Risk of depression by quartile of dietary inflammatory index stratified according to BMI (*N* = 1,865).

	**Participants without depression *N* (%)**	**Participants with depression *N* (%)**	**Model 1**	**Model 2**	***P_Interaction_***
			**OR (95% CI)**	**OR (95% CI)**	
BMI <18.50 kg/m2 (*N =* 55)			1.06 (0.88–1.15)	1.02 (0.82–1.12)	<0.001
For 1-SD increase	*N =* 42	*N =* 13	Reference	Reference	
Q1	10 (23.8%)	3 (23.1%)	0.94 (0.78–1.28)	0.98 (0.85–1.35)	
Q2	12 (28.6%)	5 (38.5%)	1.02 (0.85–1.32)	1.05 (0.86–1.37)	
Q3	10 (23.8%)	3 (23.1%)	0.98 (0.81–1.36)	1.12 (0.92–1.41)	
Q4	10 (23.8%)	2 (15.4%)	1.16 (0.90–1.42)	1.18 (0.98–1.48)	
BMI <24 kg/m2 (*N =* 700)					
For 1-SD increase	*N =* 543	*N =* 158	1.10 (0.88–1.25)	1.08 (0.86–1.20)	
Q1	117 (21.5%)	47 (29.7%)	Reference	Reference	
Q2	141 (26.0%)	51 (32.3%)	0.98 (0.81–1.17)	1.11 (0.94–1.26)	
Q3	154 (28.4%)	32 (20.3%)	1.05 (0.90–1.22)	0.98 (0.85–1.28)	
Q4	130 (23.9%)	28 (17.7%)	1.08 (0.94–1.28)	1.17 (0.96–1.36)	
24 ≤ BMI <28 kg/m2 (*N =* 754)					
For 1-SD increase	*N =* 574	*N =* 180	1.06 (0.96–1.15)	1.06 (0.86–1.15)	
Q1	144 (25.1%)	49 (27.2%)	Reference	Reference	
Q2	140 (24.4%)	46 (25.6%)	1.02 (0.89–1.17)	1.02 (0.92–1.18)	
Q3	142 (24.7%)	36 (20.0%)	1.08 (0.91–1.21)	1.08 (0.98–1.21)	
Q4	148 (25.8%)	49 (27.2%)	1.11 (0.96–1.28)	1.13 (1.00–1.26)	
BMI ≥28 kg/m2 (*N =* 356)					
For 1-SD increase	*N =* 260	*N =* 96	1.12 (1.05–1.23)	1.13 (1.08–1.22)	
Q1	78 (30.0%)	19 (19.8%)	Reference	Reference	
Q2	50 (19.2%)	23 (24.0%)	1.09 (1.03–1.18)	1.08 (1.04–1.18)	
Q3	62 (23.8%)	25 (26.0%)	1.13 (1,05–1.28)	1.16 (1.08–1.28)	
Q4	70 (26.9%)	29 (30.2%)	1.27 (1.12–1.39)	1.27 (1.12–1.42)	

*Model 1: unadjusted. Model 2: age, sex, physical activity, education level, smoking status, drinking status, hypertension, diabetes, daily energy intake, employment status. Q, quartile. P_Interaction_ was calculated using the multiplicative interaction term (E-DII × BMI)*.

### Mediating Role of BMI

**Figure 3:**
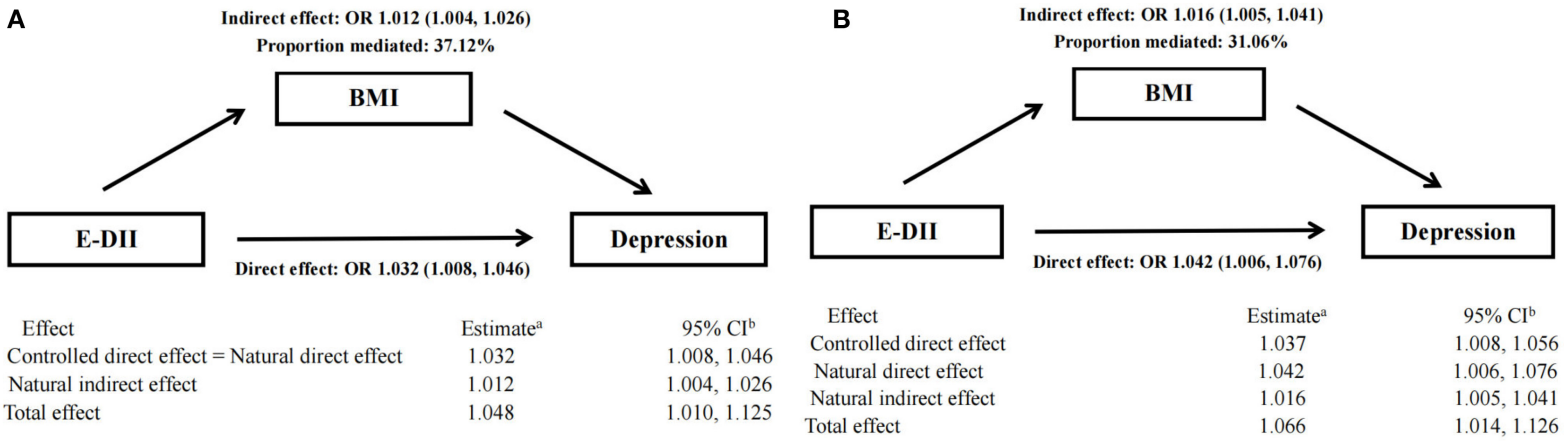
Mediation analyses without **(A)** and with **(B)** an interaction between DII and BMI on type depression risk.a Logistic model adjusted for age, sex, physical activity, education level, smoking status, drinking status, daily energy intake, and employment status. bThe 95% CI of these estimates was computed using the bootstrap method (1,000 samples).

The result of mediation analysis is shown in Figure 3. First, we hypothesized a simple mediation model without E-DII × BMI interaction on the risk of depression. Increased E-DII is associated with increased risk of depression, and the effect (37.12%) can be explained by a significant indirect effect of BMI (NIE: OR: 1.012 [95% CI: 1.004, 1.026], shown in Figure 3A). Then, based on the above-mentioned interaction between E-DII and BMI on depression risk, we used a mediation model that allows exposure–media interaction. According to this model, BMI represents both a mediator and an effect modifier of the relationship between E-DII and depression. Increased E-DII is also associated with increased risk of depression (NDE and NIE OR: 1.042 [95% CI: 1.006, 1.076] and OR: 1.016 [95% CI: 1.005, 1.041], respectively), but the proportion explained by the indirect influence of BMI was low (31.06%, shown in Figure 3B).

## Discussion

Based on the baseline data among 1,865 elderly people in the CCSNSD cohort, we found a positive non-linear relationship between the baseline inflammatory characteristics of the diet and the risk of depression, and this relationship is independent of most known or potential risk factors or confounding factors. Participants with higher DII (corresponding to the increased pro-inflammatory potential of the diet) have a higher risk of depression compared with those having lower DII (corresponding to an anti-inflammatory diet). This association is partly mediated by BMI and its interaction with DII.

To the best of our knowledge, our work is the first prospective study that showed a positive non-linear relationship between the pro-inflammatory potential of diet and the incidence of depression. Although the Geriatric Depression Scale (GDS) is currently widely used in the evaluation of depressive symptoms in elderly people older than 65 years old, we compared the baseline characteristics of the two groups (55 ≤ age ≤ 65 years vs. age>65 years) and the incidence of depressive symptoms, and the results showed that there was no significant difference between the two groups (Supplementary Table 1), so the study population is limited to adults 55 years and older.

Pro-inflammatory diet is associated with adverse mental health and mental disorders (21, 22). The meta-analysis showed that the pro-inflammatory diet estimated using the higher DII score was independently associated with increased risk of depression (23). In the Mediterranean population, a pro-inflammatory diet is associated with a considerably higher risk of depression. This association is strong in elderly subjects and subjects with cardiometabolic diseases (24). In the elderly population in the United States, a pro-inflammatory diet may be associated with a higher incidence of depressive symptoms (25). The inflammatory properties of diet act in regulating inflammation of adipose tissue in adults (26). A positive correlation was observed between obesity (BMI ≥ 30 kg/m^2^) and depression (27). Patients with a higher initial increase in BMI showed a greater decrease in depression severity. The underlying mechanism remains unclear, thus requiring further research (28). The increase in body weight and BMI is associated with a decrease in symptoms and functional improvement at the end of depression. Depression and obesity should be treated at the same time to optimize the clinical outcome of depression treatment (29). Although the Geriatric Depression Scale (GDS) is currently widely used in the evaluation of depressive symptoms in elderly people older than 65 years old, we compared the baseline characteristics of the two groups and the incidence of depressive symptoms, and the results showed that there was no significant difference between the two groups, so we The study population is limited to adults 55 years and older.

The results of the present study show that both the direct influence of DII and the indirect influence of BMI are intermediary are risk factors for the elderly suffering from depression. In the past few decades, the role of diet in regulating inflammation has received intense attention, and DII has been widely used to assess the potential of dietary inflammation. A significant association has been observed between a high pro-inflammatory diet assessed by a higher DII score and depression (13, 30, 31). However, limited studies have evaluated the mechanism of the association between inflammation and depression. Inflammation and neurodegenerative processes play an important role in depression, and the neurodegenerative exacerbation of depression may be caused at least in part by the inflammatory process. The findings on various inflammatory factors, oxygen free radical damage, tryptophan catabolites, and neurodegenerative biomarkers in patients with depression have also been confirmed by animal models of depression (32). Therefore, a high pro-inflammatory diet may increase the inflammatory process in the body and increase the risk of depression.

Depression is one of the most common diseases in the world and is closely related to chronic, low-grade inflammation. Inflammation is a pivotal and crucial mediator of the relationship between obesity and depression (33). Obesity (≥28 kg/m^2^) increases the circulating pro-inflammatory cytokines, and cytokines raised during obesity under chronic stress promote depression through several behaviors, including disruption of neurotransmitter synthesis and signal transduction (34). Moreover, diet-induced inflammation is significantly associated with the risk of overweight adults with the rs9939609 polymorphism of the Fat Mass and Obesity Associated (FTO) gene. According to diet self-reports, a normal weight does not necessarily indicate that the diet has a low inflammatory potential and good quality (35, 36). Furthermore, obesity deteriorates the psychological health of individuals. Studies have found associations between obesity and mental disorders, such as anxiety or depressive disorders (37–39). Stress induces microglia activation and neuroinflammation, which plays a vital role in the pathogenesis of depression. Peroxisome proliferator-activated receptorγ (PPARγ) is a nuclear transcription factor that can regulate microglia polarization and neuroinflammation. PPARγ mediates the microglia activation phenotype, which may be related to the susceptibility of stressed ob/ob mice to depression (40). In the indirect effect mediated by BMI, high pro-inflammatory diet is also a risk factor for GDS, and it accounts for 34.16% of the total effect. Some studies have shown higher BMI and predicted more inflammatory cytokines. Inflammatory cytokines in healthy subjects are related to BMI, but it is offset by the compensatory increase in anti-inflammatory cytokines, thereby reducing the total inflammatory burden caused by higher BMI (41, 42).

The intake of macronutrients in patients with depression is different, and the intake of these abnormal macronutrients may be associated with the development of depression. The 2019 Lancet Psychiatry Commission concluded that people with depression have a higher risk of premature death and morbidity due to unhealthy food choices, the adverse effects of certain treatments, and the impact of symptoms (43). Therefore, identifying advanced areas of knowledge to reduce mortality and morbidity among this vulnerable population is becoming a health priority. Studies have shown that patients with depression report eating more energy and energy-producing macronutrients (such as carbohydrates, protein, and fat) (44). At the same time, vitamin D from solar ultraviolet B appears to be statistically significantly reduced in patients with depression (45). What's more, sleep changes are a common problem for people with depression. In fact, in many cases, it is the main complaint. In the past, sleep problems have been viewed as a symptom or result of depression or its treatment. In recent years, more and more evidences have shown that sleep problems are comorbidities and are contributing or exacerbating factors in the onset of depression (46). There have been studies that the sleep quality of healthy adults is positively correlated with magnesium intake and negatively correlated with vitamin B12 (47).

To the best of our knowledge, the current study is the first to explore the role of BMI in the association between the DII and the risk of depression among the elderly in Northern China. We adjusted our analyses for numerous depression risk factors as potential confounders. This study has some limitations. First, among the 45 food parameters, only 22 food parameters can be used in the DII calculations, and the estimation of the possibility of dietary inflammation may have deviations. Second, considering that all participants recruited into the cohort are from the same province, the true state of the nation's elderly may not be accurately reflected. Lastly, the dietary consumption level is estimated based on the food frequency questionnaire (FFQ) covering the past 12 months, which may have a certain recall bias.

## Conclusion

In summary, our results indicate that a diet with a high pro-inflammatory potential is associated with a higher risk of depression in the elderly, and obesity as assessed by BMI here is one of the main mediators that may occur. These results help to improve our understanding of the mechanisms of diet-related inflammation and depression in the elderly. In addition, our findings support current diet-based methods to prevent depression in the elderly.

## Data Availability Statement

The original contributions presented in the study are included in the article/Supplementary Material, further inquiries can be directed to the corresponding author/s.

## Ethics Statement

The studies involving human participants were reviewed and approved by the National Institute for Nutrition and Health, Chinese Center for Disease Control and Prevention. The patients/participants provided their written informed consent to participate in this study.

## Author Contributions

YM and RL: Had full access to all data in the study, take responsibility for the integrity of the data and the accuracy of the data analysis, and concept and design. All authors: Acquisition, analysis, or interpretation of data, and critical revision of the manuscript for important intellectual content. YM, RL, and WZ: Drafting of the manuscript. RL, WZ, YM, ZZ, XJ, and XH: Statistical analysis. YM and XH: Obtained funding. XH, JW, and SL: Administrative, technical, or material support. YM: Supervision. All authors contributed to the article and approved the submitted version.

## Funding

This project was funded by the Ministry of Science and Technology of the People's Republic of China (Grant Number: 2017YFC0907701).

## Conflict of Interest

The authors declare that the research was conducted in the absence of any commercial or financial relationships that could be construed as a potential conflict of interest.

## Publisher's Note

All claims expressed in this article are solely those of the authors and do not necessarily represent those of their affiliated organizations, or those of the publisher, the editors and the reviewers. Any product that may be evaluated in this article, or claim that may be made by its manufacturer, is not guaranteed or endorsed by the publisher.
