# Supplementary material for: Role of BMI in the Relationship Between Dietary Inflammatory Index and Depression: An Intermediary Analysis
Source: Front Med (Lausanne). 2021 Nov 15;8:748788. doi: 10.3389/fmed.2021.748788 (PMC8634657; doi:10.3389/fmed.2021.748788)
Supplement: Supplementary file 1 [file Table_1.DOC]

[S](../../../../C:/Program%20Files/WindowsApps/NeteaseYoudao.18692F27B7C6F_0.0.7.0_x64__7x355j7kq8bfj/VFS/Local%20AppData/youdao/dict/Application/0.0.7.0/resultui/html/index.html#/javascript:;)upplementary Table 1 Baseline characteristics of the study population(CCSNSD cohort data, N=1865)

|  | 55 years≤Age≤65 years（n=1004） | Age＞65 years（n=861） | P-value |
| --- | --- | --- | --- |
| Sex |  |  | 0.642 |
| Male | 388(38.6%) | 328(38.1%) |  |
| Female | 616(61.4%) | 533(61.9%) |  |
| BMI（kg/m2） |  |  | 0.508 |
| BMI<18.50 (underweight) | 31(3.1%) | 24(2.8%) |  |
| 18.5≤BMI<24.00 (normal weight) | 365(36.4%) | 336(39.0%) |  |
| 24.00≤BMI<28.00 (overweight) | 422(42.0%) | 332(38.6%) |  |
| BMI≥28.00 (obese) | 186(18.5%) | 169(19.6%) |  |
| Employment |  |  | 0.176 |
| No | 871(86.8%) | 738(85.7%) |  |
| Yes | 133(13.2%) | 123(14.3%) |  |
| Education |  |  | 0.208 |
| Illiteracy | 258(25.7%) | 213(24.7%) |  |
| Primary school | 330(32.9%) | 276(32.1%) |  |
| Junior high school/above | 416(41.4%) | 372(43.2%) |  |
| Daily energy intake (kcal) |  |  | 0.815 |
|  | 1445.12±12.26 | 1448.52±10.62 |  |
| Tobacco Smoking |  |  | 0.187 |
| No | 854(85.1%) | 721(83.7%) |  |
| Yes | 150(14.9%) | 140(16.3%) |  |
| Alcohol Drinking |  |  | 0.092 |
| No | 916(91.2%) | 773(89.8%) |  |
| Yes | 88(8.8%) | 88(10.2%) |  |
| Physical activities |  |  | 0.216 |
| No | 610(60.8%) | 527(61.2%) |  |
| Yes | 394(39.2%) | 334(38.8%) |  |
| Hypertension |  |  | 0.812 |
| No | 405(40.3%) | 351(40.8%) |  |
| Yes | 599(59.7%) | 510(59.2%) |  |
| Diabetes |  |  | 0.519 |
| No | 841(83.8%) | 724(84.1%) |  |
| Yes | 163(16.2%) | 137(15.9%) |  |
| Depression |  |  | 0.618 |
| No | 768(76.5%) | 650(75.5%) |  |
| Yes | 236(23.5%) | 211(24.5%) |  |

Data are N (%) for categorical variables

Data are mean (SEM) for continuous variables
